# Identification and Validation of a Prognostic Prediction Model in Diffuse Large B-Cell Lymphoma

**DOI:** 10.3389/fendo.2022.846357

**Published:** 2022-04-14

**Authors:** Jiaqin Yan, Wei Yuan, Junhui Zhang, Ling Li, Lei Zhang, Xudong Zhang, Mingzhi Zhang

**Affiliations:** ^1^ Department of Oncology, The First Affiliated Hospital of Zhengzhou University, Zhengzhou, China; ^2^ The Academy of Medical Sciences, Zhengzhou University, Zhengzhou, China; ^3^ State Key Laboratory of Esophageal Cancer Prevention and Treatment, Zhengzhou University, Zhengzhou, China; ^4^ Otorhinolaryngology, The Third Affiliated Hospital of Zhengzhou University, Zhengzhou, China

**Keywords:** prediction model, immune cell infiltration, nomogram, stratification analyses, diffuse large B-cell lymphoma

## Abstract

**Background:**

Diffuse large B-cell lymphoma (DLBCL) is a heterogeneous group with varied pathophysiological, genetic, and clinical features, accounting for approximately one-third of all lymphoma cases worldwide. Notwithstanding that unprecedented scientific progress has been achieved over the years, the survival of DLBCL patients remains low, emphasizing the need to develop novel prognostic biomarkers for early risk stratification and treatment optimization.

**Method:**

In this study, we screened genes related to the overall survival (OS) of DLBCL patients in datasets GSE117556, GSE10846, and GSE31312 using univariate Cox analysis. Survival-related genes among the three datasets were screened according to the criteria: hazard ratio (HR) >1 or <1 and *p*-value <0.01. Least Absolute Shrinkage and Selection Operator (LASSO) and multivariate Cox regression analysis were used to optimize and establish the final gene risk prediction model. The TCGA-NCICCR datasets and our clinical cohort were used to validate the performance of the prediction model. CIBERSORT and ssGSEA algorithms were used to estimate immune scores in the high- and low-risk groups.

**Results:**

We constructed an eight-gene prognostic signature that could reliably predict the clinical outcome in training, testing, and validation cohorts. Our prognostic signature also performed distinguished areas under the ROC curve in each dataset, respectively. After stratification based on clinical characteristics such as cell-of-origin (COO), age, eastern cooperative oncology group (ECOG) performance status, international prognostic index (IPI), stage, and MYC/BCL2 expression, the difference in OS between the high- and low-risk groups was statistically significant. Next, univariate and multivariate analyses revealed that the risk score model had a significant prediction value. Finally, a nomogram was established to visualize the prediction model. Of note, we found that the low-risk group was enriched with immune cells.

**Conclusion:**

In summary, we identified an eight-gene prognostic prediction model that can effectively predict survival outcomes of patients with DLBCL and built a nomogram to visualize the perdition model. We also explored immune alterations between high- and low-risk groups.

## Introduction

Lymphoma is the fourth most common cancer and the sixth leading cause of cancer death in the United States (1). Diffuse large B-cell lymphoma (DLBCL) accounts for approximately one-third of all lymphoma cases worldwide (2–4). In the current World Health Organization (WHO) lymphoma classification, about 80% of DLBCL cases are designated as not otherwise specified (NOS) (2). Three molecularly distinct forms of DLBCL have been identified by gene expression patterns, specifically an activated B cell-like (ABC) and germinal center B-cell-like (GCB) types and a small amount were unclassified DLBCL (UC) (5–7). Lymphomas with rearrangements of MYC with BCL2 and/or BCL6 are called “double-hit lymphomas”(DHL) or “triple-hit lymphomas”(THL) (8). There also exist one subtype called “double-expressor lymphomas” (DELs), defined as co-expression of MYC and BCL2 (9). DLBCL comprises a heterogeneous group with pathophysiological, genetic and clinical features (4). Albeit significant efforts have been made to better understand lymphomas, the overall survival (OS) of DLBCL patients remains dismal (5, 10). Accordingly, developing novel prognostic biomarkers for early risk stratification and treatment optimization is imperative.

It is well established that clinical prognosis systems for DLBCL, including the rituximab international prognostic index (IPI), age-adjusted IPI, and NCCN-IPI, use clinical factors for risk stratification of patients (4). Although IPI is easy to apply during clinical practice, it does not fully account for disease heterogeneity (11, 12). An increasing body of evidence suggests that patients with the ABC disease subtype have significantly poorer outcomes with standard up-front rituximab-containing chemoimmunotherapy than patients with GCB disease (13). A survival-related gene prognostic model, in combination with other prognostic indicators such as IPI and cell of origin (COO), might improve our assessment of patient prognosis for individualized treatment.

It is widely acknowledged that the tumor microenvironment (TME) of patients with lymphoma comprises endothelial cells, fibroblasts, adipocytes, and immune cells and is a key factor for tumor initiation and metastasis (14, 15). Several studies have focused on the potential role of the TME, especially the immune status in DLBCL pathogenesis (16, 17). Therefore, it is critical to better characterize the TME to develop the treatments for DLBCL patients (18).

Thanks to high-throughput genome sequencing technique, there had been several studies exploring potential prognostic biomarkers of DLBCL patients on genomic level. Xie et al. for example, investigated the prognostic value of m6A regulators and established an m6A-based prognostic gene signature for DLBCL (19). Feng et al. constructed a 14-gene prognostic signature deriving from immune-related genes for 216 DLBCL patients (20). Luo et al. identified the aging-related genes associated with prognostic value in DLBCL patients (21). In this study, we integrated the transcriptome data from the Gene Expression Omnibus (GEO), The Cancer Genome Atlas (TCGA), and our clinical cohort and constructed an eight-gene signature-based prediction model. Furthermore, we explored the immune alterations in high- and low-risk groups.

## Methods and Materials

### Collection of Clinical DLBCL Specimens

DLBCL specimens were obtained through biopsy in the First Affiliated Hospital of Zhengzhou University and frozen at −80°C for storage. All participants provided written informed consent for the use of their specimens in this study. Clinicopathological features of 45 DLBCL patients from the First Affiliated Hospital of Zhengzhou University are performed in **Supplementary Table 1**. The study protocol was approved by the ethics committee of the First Affiliated Hospital of Zhengzhou University (ethics number 2021-KY-0835-001).

### Selection of DLBCL Gene Expression Datasets

We systematically explored publicly available DLBCL gene expression datasets with corresponding clinical information of patients from the GEO (https://www.ncbi.nlm.nih.gov/geo/) and TCGA (https://portal.gdc.cancer.gov/) databases. For this study, we gathered a total of 2,335 patients with DLBCL from four cohorts, including GSE117556 (*n* = 928), GSE10846 (*n* = 420), GSE31312 (*n* = 498), and TCGA-NCICCR (*n* = 489) (**Table 1**) and our clinical cohort (*n* = 45). Patients with incomplete transcriptomic data and clinical data were excluded. Ultimately, we included 928 patients from GSE117556, 414 from GSE10846, 470 from GSE31312, and 234 from TCGA-NCICCR. The GSE10846 and GSE31312 datasets used the GPL570 platform, while the GSE117556 dataset used the GPL14951 platform.

**Table 1:** Characteristics of the included datasets.

Dataset ID	Country	Number of samples	GPL ID	Number of rows per platform
GSE117556	UK	928	GPL14951	29,377
GSE10846	USA	420	GPL570	54,675
GSE31312	USA	498	GPL570	54,675
TCGA-NCICCR	USA	489	NA	56,753
Clinical cohort	China	45	NA	NA

GSE, Gene Expression Omnibus Series; GPL, Gene Expression Omnibus Platform; UK, United Kingdom; USA, United States of America; NA, Not Available.

### Construction and Validation of Prediction Model

In this study, we used univariate Cox analysis to screen genes related to the OS of DLBCL patients in the GSE117556, GSE10846, and GSE31312 datasets. Genes with hazard ratio (HR) >1 and HR <1 were defined as the risk and protective genes. A *p*-value <0.01 was the cutoff point. The risk genes and protective genes shared by the three datasets were intersected and combined with Least Absolute Shrinkage and Selection Operator (LASSO) regression and multivariate Cox regression to build the final gene risk prediction model (22). We conducted univariate and multivariate Cox regression analyses using the R package “survival.” Another R package “glmnet” was used for the LASSO Cox regression analysis (23). The risk score (RS) of each sample was calculated by multivariate Cox regression analysis. The correlation analysis was based on the R package “corrplot,” and the forest plots for univariate and multivariate analyses were constructed by R package “forestplot.”

### TME Characterization Analysis

We employed two algorithms to assess immune infiltration in DLBCL. CIBERSORT (http://cibersort.stanford.edu/) algorithm was used to obtain the proportion of 22 immune cell types with a threshold of *p* < 0.05 (24). We applied Single-Sample Gene Set Enrichment Analysis (ssGSEA) to assess the infiltration level of 28 different immune cells in DLBCL expression profile data by the “GSVA” package (25). The R package “ggpubr” was used to visualize differences in the distributions of immune-related cells in the low- and high-risk patient groups from the overall cohort. ”^***^,” “^**^,” “^*^,” and “ns” indicate *p* < 0.001, *p* < 0.01, *p* < 0.05, and not significant, respectively, for the Kruskal–Wallis test.

### Quantitative Real-Time Polymerase Chain Reaction

Total RNA was isolated from the specimens harvested using TRIzol reagent (Invitrogen Corporation, Carlsbad, CA, # A33250). The Prime Script RT reagent kit with genomic DNA eraser (TaKaRa, Tokyo, Japan, #RR037A) was used to synthesize complementary DNA (cDNA). Quantitative real-time polymerase chain reaction (qRT-PCR) analyses were detected by SYBR Green Master Mix (TaKaRa), and the primers for qRT-PCR analyses are listed in **Supplementary Table 2**. The 2^−ΔCT^ method was utilized to calculate the relative mRNA expression of each gene.

### Statistical Analysis

Statistical analyses were performed with R (version 3.6.3). The Kaplan–Meier method was used to assess the differences in survival time, and the log-rank test was used to determine the statistical significance. Time-dependent receiver operating characteristic (ROC) curve analysis was used to measure the prognostic performance by comparing the areas under curves (AUC). The nomogram was plotted using the “rms” package (26). The difference in immune infiltration levels between high- and low-risk groups was calculated by Kruskal–Wallis test, and a *p*-value < 0.05 was statistically significant.

## Results

### Identification and Validation of a Prognostic Signature

**Figure 1:**
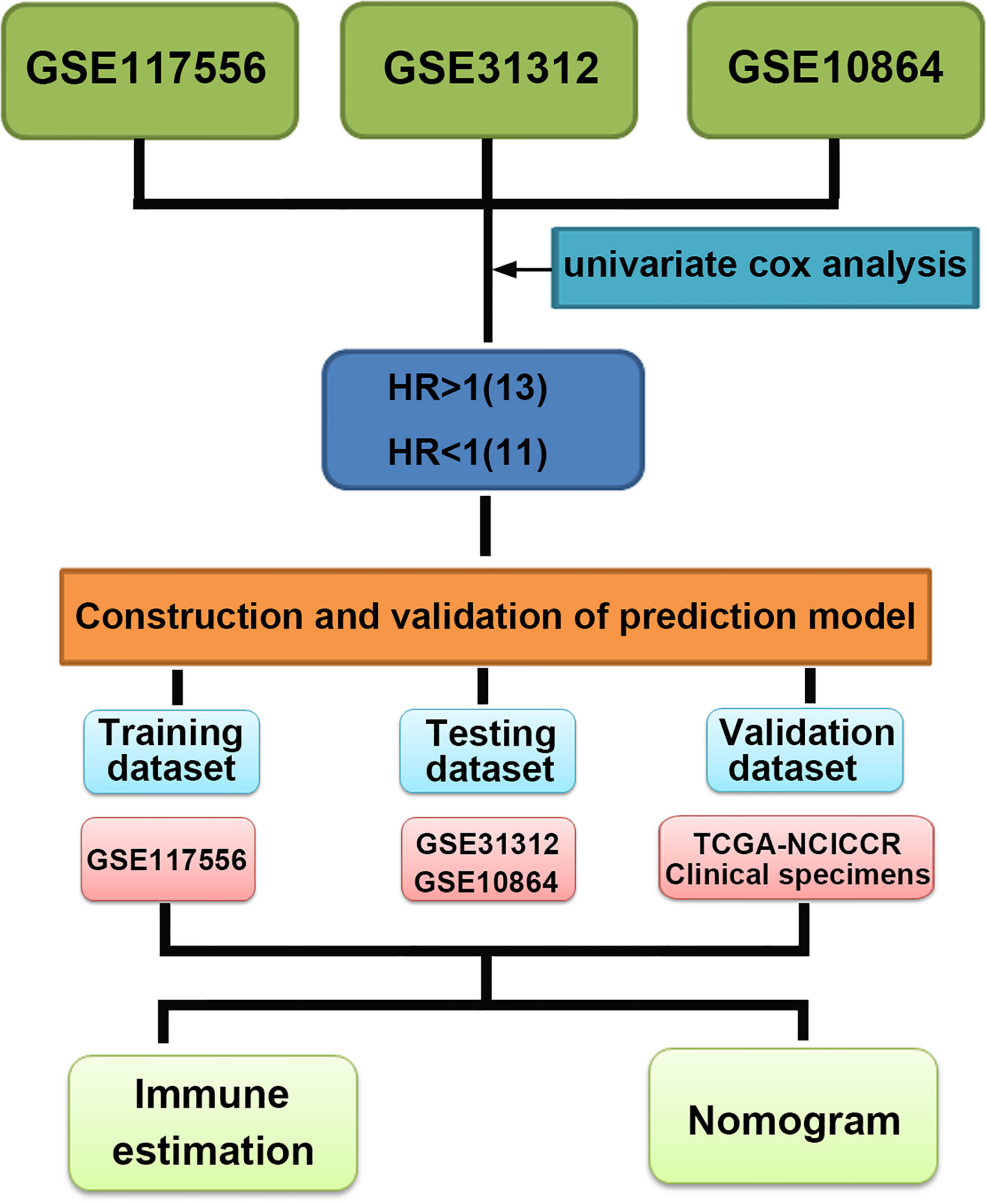
Multistep analysis of the study.

**Figure 2:**
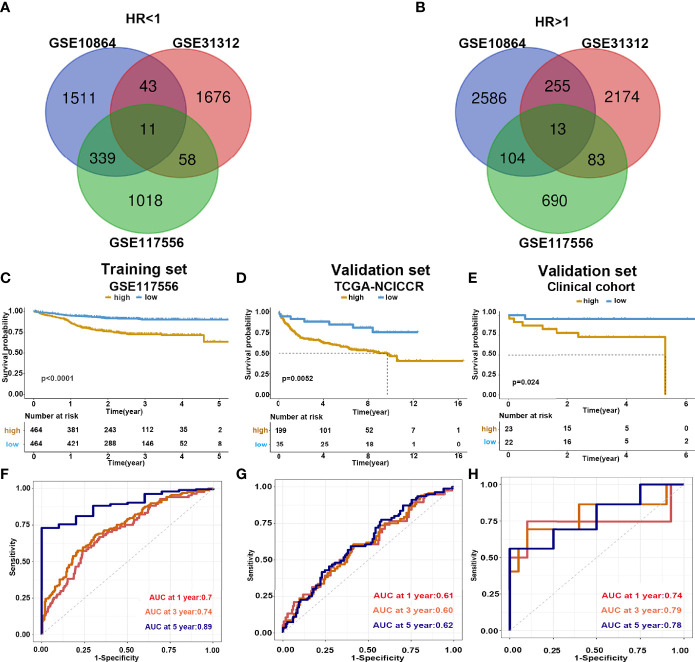
Construction and validation of prediction model. **(A, B)** Venn diagram shows the common protective and risk genes identified by three datasets. **(C–E)** Kaplan–Meier analysis for the eight-gene signature in the GSE117556, TCGA-NCICCR, and our clinical cohort, respectively. **(F–H)** Time-dependent ROC curve analysis of 1, 3, and 5 years in GSE117556, TCGA-NCICCR, and our clinical cohort.

To identify the prognostic signature of DLBCL, we performed a multiple-step analysis (**Figure 1**). We first screened the GEO database and selected three datasets for univariate Cox proportional hazards regression to identify candidate genes significantly related to OS. We conducted univariate Cox analysis in the GSE117556, GSE10846, and GSE31312 datasets with *p* < 0.01 as the cutoff value. In total, 1,426, 1,904, and 1,788 candidate protective genes (with hazard ratios (HR) <1) and 890, 2,958, and 2,525 candidate risk genes (with HR >1) were identified in GSE117556, GSE10846, and GSE31312, respectively. A total of 11 genes were candidate protective genes after intersecting the candidate protective genes (**Figure 2A**). Similarly, after matching the candidate risk genes identified in the three datasets, 13 common genes are retained (**Figure 2B**).

The detailed information on these genes is listed in **Table 2**. Next, GSE117556 was assigned as the training dataset, and the LASSO was applied to screen the candidate genes, yielding eight genes (**Supplementary Figures 1A, B**). Ultimately, the HRs of the eight genes were acquired by conducting multivariate Cox regression analysis. The forest plot (**Supplementary Figure 1C**) showed that HK2, GAB1, GRPEL1, RCSD1, PLAC8L1, and RASAL1 were risk factors (HR >1), and CAPG and PDPN were protective factors (HR <1) for OS. Finally, the following risk score model was established: risk score = 0.271 × HK2 expression + 0.182 × GAB1 expression + 0.172 × RASAL1 expression − 0.254 × CAPG expression − 0.358 × PDPN expression + 0.362 × GRPEL1 expression + 0.370 × RCSD1 expression + 0.177 × PLAC8L1 expression. Each patient was assigned a risk score with the prognostic model. According to the median risk score, patients in the GSE117556 dataset were stratified into high-and low-risk groups. Of the eight-gene prognostic signature, HK2 have the most significant correlation with worse survival. Through analyzing the RT-PCR results deriving from our clinical specimens, We found HK2 had high expressed in high stage DLBCL (**Supplementary Figure 2A**). To further explore the function of HK2, we first identified genes that correlated with HK2 in the GSE117556 dataset (**Supplementary Figure 2B**). We then utilized positive and negative correlated genes to perform GO and KEGG enrichment analyses. Results revealed that positive correlated genes were involved in cell cycle (**Supplementary Figure 2C**) and negative correlated genes were involved in immune response (**Supplementary Figure 2D**). These results partly reflect genomic differences between high- and low-risk groups.

**Table 2:** Detail information of selected common genes in three datasets.

Gene symbol	Gene name	Datasets (HR/*p*-value)		
		GSE117556	GSE31312	GSE10864
COL1A1	Collagen type I alpha 1 chain	0.763 (*p* < 0.001)	0.043 (*p* = 0.009)	0.829 (*p* = 0.004)
ST6GALNAC5	ST6N-Acetylgalactosaminide alpha-2,6-sialyltransferase 5	0.727 (*p* < 0.001)	0.184 (*p* = 0.009)	0.796 (*p* < 0.001)
CAPG	Capping actin protein, gelsolin like	0.728 (*p* < 0.001)	0.145 (*p* < 0.001)	0.793 (*p* < 0.001)
LRRC15	Leucine-rich repeat containing 15	0.656 (*p* < 0.001)	0.166 (*p* = 0.001)	0.790 (*p* < 0.001)
PDPN	Podoplanin	0.656 (*p* < 0.001)	0.214 (*p* = 0.005)	0.841 (*p* < 0.001)
NEK6	NIMA-related kinase 6	0.610 (*p* < 0.001)	0.205 (*p* = 0.004)	0.660 (*p* < 0.001)
PTPN14	Protein tyrosine phosphatase nonreceptor type 14	0.738 (*p* = 0.002)	0.183 (*p* = 0.002)	0.813 (*p* < 0.001)
LOX	Lysyl oxidase	0.656 (*p* = 0.008)	0.161 (*p* = 0.001)	0.862 (*p* < 0.001)
RBP5	Retinol-binding protein 5	0.754 (*p* = 0.006)	0.215 (*p* = 0.001)	0.870 (*p* = 0.005)
NRP2	Neuropilin 2	0.747 (*p* = 0.002)	0.237 (*p* = 0.003)	0.773 (*p* = 0.003)
DST	Dystonin	0.792 (*p* = 0.005)	0.042 (*p* < 0.001)	0.748 (*p* = 0.001)
MSL1	MSL complex subunit 1	1.592 (*p* = 0.004)	12.721 (*p* < 0.001)	1.459 (*p* = 0.002)
GRPEL1	GrpE-like 1, mitochondrial	1.797 (*p* < 0.001)	4.534 (*p* = 0.008)	1.877 (*p* < 0.001)
RCSD1	RCSD domain-containing 1	1.807 (*p* < 0.001)	8.005 (*p* < 0.001)	1.300 (*p* < 0.001)
PLAC8L1	PLAC8-like 1	1.277 (*p* = 0.004)	5.033 (*p* = 0.001)	1.449 (*p* < 0.001)
PRC1	Protein regulator of cytokinesis 1	1.745 (*p* = 0.001)	3.910 (*p* = 0.005)	1.330 (*p* = 0.001)
RASAL1	RAS protein activator-like 1	1.368 (*p* < 0.001)	3.056 (*p* = 0.007)	1.220 (*p* = 0.001)
LARS	Leucyl-TRNA synthetase 1	2.088 (*p* = 0.001)	5.419 (*p* = 0.007)	1.599 (*p* < 0.001)
SNHG7	Small nucleolar RNA host gene 7	1.238 (*p* = 0.006)	4.498 (*p* = 0.002)	1.439 (*p* < 0.001)
WDR12	WD repeat domain 12	1.812 (*p* = 0.001)	8.049 (*p* = 0.001)	1.526 (*p* = 0.003)
PI4K2B	Phosphatidylinositol 4-kinase type 2 beta	1.637 (*p* = 0.004)	3.868 (*p* = 0.004)	1.537 (*p* = 0.006)
MMACHC	Metabolism of cobalamin-associated C	1.430 (*p* < 0.001)	5.033 (*p* = 0.009)	1.770 (*p* < 0.001)
HK2	Hexokinase 2	1.320 (*p* = 0.004)	4.455 (*p* < 0.001)	1.438 (*p* < 0.001)
GAB1	GRB2-associated binding protein 1	1.277 (*p* = 0.008)	2.634 (*p* = 0.007)	1.235 (*p* = 0.008)

To further verify the predicting model, we analyzed the survival of high- and low-risk groups. Patients in the low-risk group demonstrated a longer survival time than those in the high-risk group (**Supplementary Figure 3**). Consistently, the Kaplan–Meier curve indicated that patients in the high-risk group had significantly worse prognoses than low-risk patients (log-rank test *p* < 0.001) (**Figure 2C**). We further validated the risk score model in the testing dataset GSE 31312 (log-rank test *p* < 0.001) (**Supplementary Figure 4A**) and GSE 10864 (log-rank test *p* < 0.001) (**Supplementary Figure 4B**), TCGA-NCICCR dataset (log-rank test *p* = 0.0052) (**Figure 2D**), and our clinical specimens (log-rank test *p* = 0.024) (**Figure 2E**), the results were consistent with the training dataset findings. The predictive power of the risk score model was assessed by time-dependent ROC, which yielded good performance in the above datasets. In dataset GSE117556, the AUC values for 1-, 3-, and 5-year overall survival were 0.7, 0.74, and 0.89, respectively. In TCGA-NCICCR dataset, the AUC values for 1-, 3-, and 5-year overall survival predictions were 0.61, 0.60, and 0.62, respectively. Our clinical cohort yielded AUC values of 0.74, 0.79, and 0.78 for the 1-, 3-, and 5-year overall survival, respectively (**Figures 2F–H**). In dataset GSE31312, the AUC values for 1-, 3-, and 5-year overall survival were 0.64, 0.7, and 0.74, respectively. In dataset GSE10864, the AUC values for 1-year overall survival were 0.56 (**Supplementary Figures 4C, D**). The above AUC curves provided an objective validation of the clinical application value of our model.

### Validation of the Accuracy of the Risk Score Model

**Figure 3:**
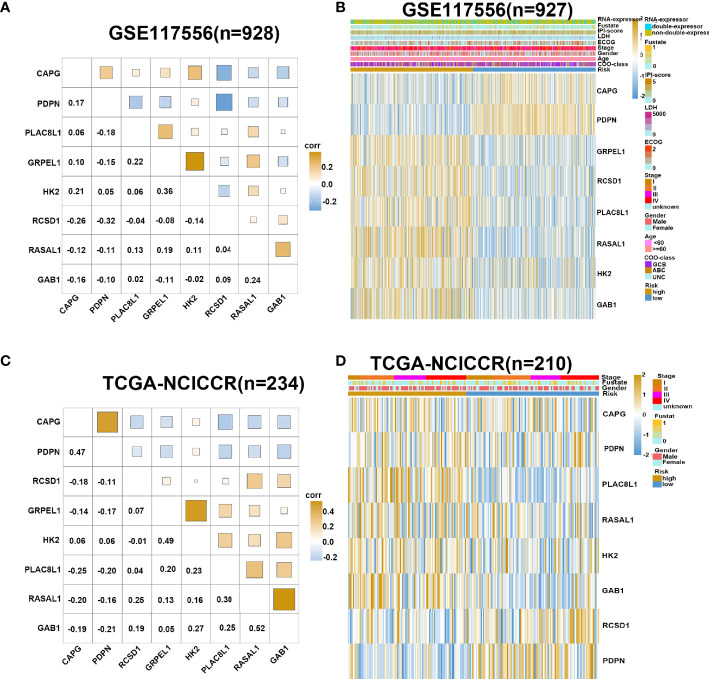
Gene expression in high- and low-risk group. **(A)** Corrplot shows correlation of eight genes in GSE117556 dataset. **(B)** Heatmap shows gene expression of eight genes and clinical parameters in high- and low-risk group in GSE117556 dataset. **(C)** Corrplot shows correlation of eight genes in TCGA-NCICCR dataset. **(D)** Heatmap shows gene expression of eight genes and clinical parameters in high- and low-risk groups in TCGA-NCICCR dataset.

We used GSE117556 as the training dataset to detect the correlation and interdependence between the eight risk genes, which proved that our prognostic signature could minimize data bias caused by gene collinearity (**Figure 3A**). Using the median risk score as the cutoff point, the training set was classified into low- and high-risk groups. Our results suggest that the risk score model has significant value for evaluating characteristic genes. The eight genes were differentially expressed between the two groups, indicating their role in contributing to the prognosis of DLBCL (**Figure 3B**). Analysis of validation dataset TCGA-NCICCR yielded consistent results (**Figures 3C, D** **)**.

### Validation of Risk Score Model Based on Different Clinical Parameters and Subgroups

**Figure 4:**
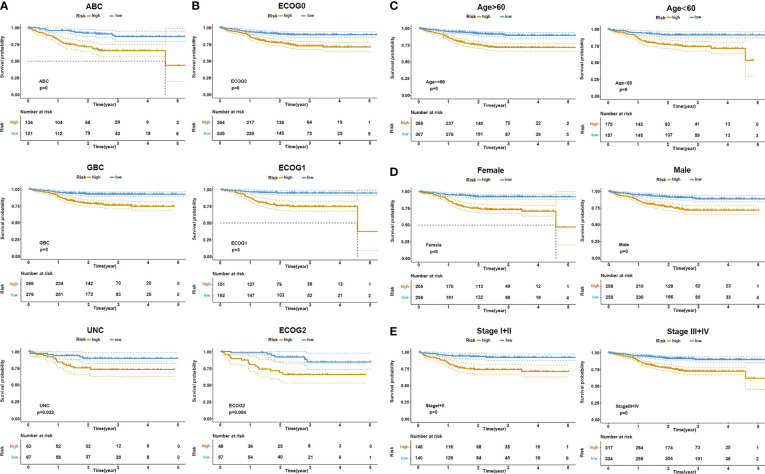
KM survival stratification analyses in the GSE117556 dataset. **(A)** COO subgroup. **(B)** ECOG stage. **(C)** Age. **(D)** Gender. **(E)** Clinical stage.

It has been established that DLBCL exhibits a significant heterogeneity in cell origin, clinical manifestations, gene expression profiles, and so on. To verify the effectiveness of the risk signature in the existing clinical subgroups, we conducted a series of subgroup analyses on dataset GSE117556. Stratification based on clinical characteristics such as COO, ECOG, MYC/BCL2 double expression, lactate dehydrogenase (LDH) value, age, stage, and gender was conducted. Among those subtypes, patients in the high-risk groups had worse survival outcomes than patients in the low-risk group (log-rank test *p* < 0.05; **Figure 4** and **Supplementary Figure 5**). These findings validated that our risk model yielded good predictive performance after stratifying for different clinicopathological characteristics.

### The Landscape of Immune Cell Infiltration in the TME of DLBCL

**Figure 5:**
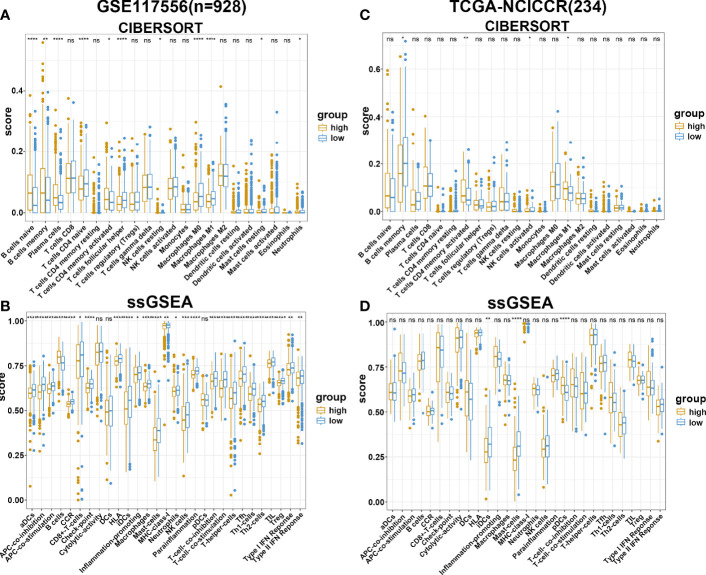
Immune estimation in high- and low-risk groups. **(A, B)** The difference of immune infiltration in high- and low-risk groups estimated by CIBERSORT and ssGSEA in the GSE117556 datasets. **(C, D)** The difference of immune infiltration in high- and low-risk groups estimated by CIBERSORT and ssGSEA in TCGA-NCICCR datasets. ns, Not Significant; * P < 0.05; ** P < 0.01; *** P < 0.001; **** P < 0.0001.

To further explore the potential survival mechanisms related to the risk score model, mRNA data from dataset GSE117556 were first used to detect the proportion of 22 immune cell types in each sample *via* the CIBERSORT algorithm. As shown in **Figure 5A**, the proportion of immune cells was significantly different between the high- and low-risk score groups. Compared with the low-risk group, the high-risk group exhibited increased B-cell infiltrations, with less-naive CD4 T cell, T follicular helper cell, M0 macrophages, M1 macrophages, and other proinflammatory cells. Consistent results were obtained when ssGSEA was applied (**Figure 5B**). In contrast with the high-risk group, tumor-infiltrating lymphocytes (TIL), antigen-presenting cells were significantly enriched in the low-risk groups. We further applied the above two algorithms to the validation dataset TCGA-NCICCR. Similar results were obtained from GSE117556 analysis results. Immune signatures between the high- and low-risk groups were different, and the low-score group was significantly infiltrated with proinflammatory immune cells (**Figures 5C, D** **)**. In addition, we analyzed immune-related genes between the two groups and further approved the above findings (**Supplementary Figure 6**).

### Validation of the Performance of Our Prediction Model

**Figure 6:**
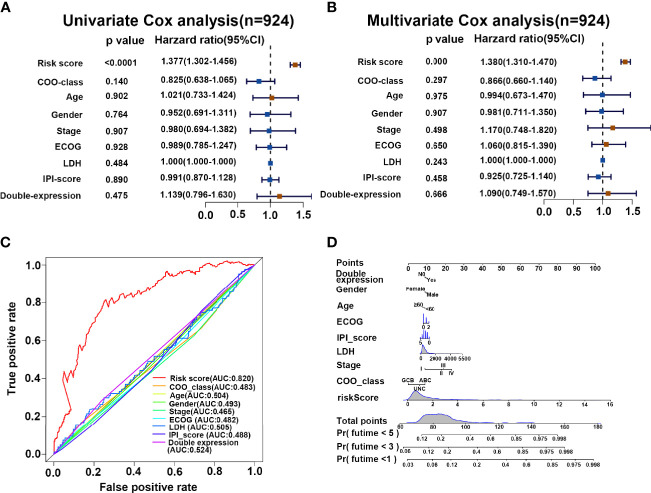
Risk score is a superior biomarker for evaluating the prognosis of DLBCL. **(A, B)** Forest plot summary of the univariate and multivariable analyses of risk score and other clinical parameters. **(C)** ROC analysis of risk score and other clinical parameters. **(D)** Nomogram integrating the risk score and clinical parameters for predicting the probability of patient mortality at 1, 3, and 5 years of OS.

To evaluate the performance of our risk score model on the prognosis of DLBCL patients, we integrated the clinicopathological characteristics with risk score signatures in different algorithms. As shown in **Figure 6A**, univariate Cox regression analysis demonstrated that the risk score model was a significant predictor of OS in patients with DLBCL (*p* < 0.0001, HR = 1.377), compared with other clinicopathological characteristics. Multivariate Cox regression analysis showed that the risk score model was an independent prognostic factor for poor prognosis (*p* < 0.0001, HR = 1.380) (**Figure 6B**). Compared with other indicators, the risk score was superior for predicting the patient prognosis (AUC = 0.820) (**Figure 6C**). Finally, we established a nomogram based on the above clinical parameters to predict patient prognosis quantitatively. Accordingly, our nomogram has huge prospects for clinical application for predicting the OS of individual DLBCL patients (**Figure 6D**).

## Discussion

Notwithstanding that unprecedented scientific progress has been achieved over the years, the survival of DLBCL patients remains relatively low. In this regard, the cure rate of DLBCL ranges from 40% to 60% following standard frontline immunochemotherapy (5). However, a poor prognosis has been reported for patients with refractory disease, those who relapse after salvage chemotherapy and autologous stem cell transplant or chimeric antigen receptor T-cell therapy, highlighting the need for novel therapeutic approaches (27, 28). Indeed, good prediction models, like good therapies, are best compared head-to-head in novel patient populations (29). Therefore, developing a novel prognostic model in combination with other prognostic indicators IPI and COO might be necessary to assess the patient prognosis for individualized treatment. In recent years, an increasing number of studies have been conducted to identify novel prognostic indicators. For instance, Schmitz et al. applied exome and transcriptome sequencing methods on 574 DLBCL biopsy samples to construct a new genetic subtype for DLBCL classification to guide therapy (30). Han et al. demonstrated that piRNA-30473, which promotes DLBCL progression by regulating m6A RNA methylation in DLBCL, can improve the prognostic stratification and therapeutic approach (31). With the development of next-generation sequencing, many prognostic cancer models have been established in recent years (32–34) based on public transcriptomic databases such as the GEO and TCGA datasets.

The present study explored DLBCL gene expression datasets with corresponding clinical information of patients from the GEO to identify candidate genes that were significantly related to OS. After performing univariate Cox proportional hazards regression analysis, 11 protective and 13 risk genes were identified. Finally, we constructed an eight-gene prognostic signature through the LASSO method and multivariate Cox regression analysis. There had been several previous studies that were consistent and corroborated with the prognostic value of our risk score model. For example, HK2, GAB1, and RASAL1 were risk genes in our model. HK2 is known to be a key metabolic enzyme by promoting glucose uptake in cells and facilitating the Warburg effect. HK2 had been explored as a major player in helping maintain the highly malignant state in many types of cancer (35–37). Bhalla et al. also provided strong support for the direct contribution of HK2 in B-cell lymphoma development and suggested that HK2 is a key metabolic driver of the DLBCL phenotype (38). GAB1, which is widely distributed in various body tissues, is capable of promoting cell proliferation, and its expression may enhance the carcinogenesis and cancer progression (39, 40). Chang et al. clarified that RASAL1 was increased in ovarian adenocarcinoma tumorous tissues and HEY cells, which correlated with poor prognosis in ovarian adenocarcinoma patients (41). Kaplan–Meier analysis demonstrated that the risk model could predict the outcome for patients with DLBCL in training, testing, and validating datasets. Similar conclusions were reached when our clinical cohort data were applied. ROC curve analysis consistently indicated the good performance of our risk model. We then conducted correlation analysis to evaluate the collinearity among the eight prognostic genes. Importantly, we found that the correlation among these genes was low, suggesting that the regression coefficients of this model were reliable and stable (42).

At present, different approaches are adopted in clinical practice to evaluate the occurrence and development of DLBCL at different levels, including gene expression patterns (ABC, GCB, UC), ECOG, IPI, DELs, LDH, age-adjusted IPI, gender, stages, and so on. Patients with the GCB subtype, for example, usually have a better prognosis than the ABC subtype (6, 43). It has been established that DELs are generally aggressive and respond poorly to currently available therapies (9, 44, 45). Moreover, IPI and age-adjusted IPI have been developed as models for predicting outcomes based on clinical factors from more than 4,000 patients (46, 47). However, despite overall improvements in DLBCL patient outcomes, 30%–40% of patients develop relapsed or refractory disease (48). In the present study, after stratification based on clinical characteristics such as gene expression patterns (ABC, GCB, UC), ECOG, IPI, DELs, LDH, age-adjusted IPI, gender, and Ann Arbor stages, the difference in OS between the high- and low-risk groups was still statistically significant. This finding suggests that our model can be combined with existing clinical parameters to reduce false positives and negatives, improve diagnostic accuracy, and provide effective treatment.

An increasing body of evidence suggests that the TME affects the prognosis of DLBCL patients. Lenz et al. analyzed gene expression in 181 pretreatment biopsy specimens derived from DLBCL patients and found that the survival of patients with DLBCL was affected by immune cells, fibrosis, and angiogenesis in the tumor microenvironment (49). Mueller et al. also demonstrated that DLBCL recruited T cells and monocytes *via* CCL5 to support B-cell survival and proliferation (50). By immunohistochemical staining, Chang et al. showed the presence of CD1a+ dendritic cells (DCs) and increased granzyme B+ T cells within tumors was associated with a favorable prognosis (51). It has been established that M1 cells play a proinflammatory and anticancer role in the TME of DLBCL, while M2 type plays an immunosuppressive role to promote cancer progression (47, 52, 53). Herein, we used two algorithms to evaluate the TME of patients with DLBCL and found that patients in the high-risk score group exhibited significant B-cell infiltration with mild infiltration of M0, M1, CD8+ T cells, and DCs. Our results suggest the presence of an immunosuppressive TME in patients from the high-risk group leading to cancer progression. Finally, we evaluated the potential value of applying our risk score model to clinical practice. Based on the results of univariate regression analysis, multivariate regression analysis, and nomogram, our model has huge prospects for application in clinical practice.

However, our study was significantly limited by its retrospective nature as DLBCL samples were from different platforms, which may be a source of sampling bias. Well-designed prospective clinical trials should be conducted in the future to highlight the role of our prediction model in DLBCL progression and metastasis.

## Conclusions

In summary, this study identified an eight-gene prognostic signature that can effectively predict DLBCL patient outcomes. The eight-gene prognostic model related to TME in combination with other prognostic indicators IPI and COO might be useful to clinicians when evaluating the prognosis of patients for individualized treatment.

## Data Availability Statement

The datasets GSE117556, GSE10846 and GSE31312 for this study can be found in the GEO (https://www.ncbi.nlm.nih.gov/geo).The datasets NCICCR for this study can be found at TCGA (https://portal.gdc.cancer.gov/repository).

## Ethics Statement

The studies involving human participants were reviewed and approved by the ethics committee of the First Affiliated Hospital of Zhengzhou University. The patients/participants provided their written informed consent to participate in this study.

## Author Contributions

MZ, JY, and LL contributed to the design and implementation of the research. WY and JZ contributed to the analysis of the results and to the writing of the manuscript. JY, LZ, and XZ designed the figures. All authors listed have made a substantial, direct, and intellectual contribution to the work and approved it for publication.

## Funding

This work was supported by the National Natural Science Foundation of China (Grant no. 81500174).

## Conflict of Interest

The authors declare that the research was conducted in the absence of any commercial or financial relationships that could be construed as a potential conflict of interest.

## Publisher’s Note

All claims expressed in this article are solely those of the authors and do not necessarily represent those of their affiliated organizations, or those of the publisher, the editors and the reviewers. Any product that may be evaluated in this article, or claim that may be made by its manufacturer, is not guaranteed or endorsed by the publisher.

## Supplementary Material

Supplementary Figure 1 Selection of genes for constructing predicting model. **(A)** LASSO coefficient profiles of 24 genes. **(B)** Partial likelihood deviance plot. **(C)** Forest plot shows the multivariable Cox regression analysis of eight genes.

Supplementary Figure 2 Exploring function of HK2. **(A)** RT-PCR analysis showing HK2 expression stage I+II and III+IV. **(B)** Correlation analysis of HK2. **(C)** GO enrichment analysis of negative and positive correlated genes. **(D)** KEGG enrichment analysis of negative and positive correlated genes.

Supplementary Figure 3 Survival information of the patients between high- and low-risk group. **(A)** The correlation of risk score and patient numbers. **(B)** The correlation of risk score and survival time.

Supplementary Figure 4 Validation of prediction model in testing set. **(A, B)** Kaplan-Meier analysis between high and low risk group in GSE31312 and GSE10864 datasets. **(C, D)** ROC curve analysis of GSE31312 and GSE10864 datasets.

Supplementary Figure 5 KM survival stratifcation analyses in GSE117556 dataset. **(A)** MYC/BCL2 expression. **(B)** LDH level.

Supplementary Figure 6 Expression of immune-related genes in high and low risk group. **(A, B)** Boxplot showed difference of immune-related genes in high and low risk group in GSE117556 and TCGA-NCICCR dataset, respectively.

## References

[1] Teras LR DeSantis CE Cerhan JR Morton LM Jemal A Flowers CR. 2016 US Lymphoid Malignancy Statistics by World Health Organization Subtypes. CA Cancer J Clin (2016) 66:443–59. doi: 10.3322/caac.21357 27618563

[2] Sukswai N Lyapichev K Khoury JD Medeiros LJ. Diffuse Large B-Cell Lymphoma Variants: An Update. Pathology (2020) 52:53–67. doi: 10.1016/j.pathol.2019.08.013 31735345

[3] Pasqualucci L Dalla-Favera R. Genetics of Diffuse Large B-Cell Lymphoma. Blood (2018) 131:2307–19. doi: 10.1182/blood-2017-11-764332 PMC5969374 29666115

[4] Liu Y Barta SK. Diffuse Large B-Cell Lymphoma: 2019 Update on Diagnosis, Risk Stratification, and Treatment. Am J Hematol (2019) 94:604–16. doi: 10.1002/ajh.25460 30859597

[5] Alizadeh AA Eisen MB Davis RE Ma C Lossos IS Rosenwald A. Distinct Types of Diffuse Large B-Cell Lymphoma Identified by Gene Expression Profiling. Nature (2000) 403:503–11. doi: 10.1038/35000501 10676951

[6] Hans C Weisenburger D Greiner T Gascoyne R Delabie J Ott G. Confirmation of the Molecular Classification of Diffuse Large B-Cell Lymphoma by Immunohistochemistry Using a Tissue Microarray. Blood (2004) 103:275–82. doi: 10.1182/blood-2003-05-1545 14504078

[7] Reddy A Zhang J Davis N Moffitt A Love C Waldrop A. Genetic and Functional Drivers of Diffuse Large B Cell Lymphoma. Cell (2017) 171:481–94.e15. doi: 10.1016/j.cell.2017.09.027 28985567 PMC5659841

[8] Aukema SM Siebert R Schuuring E van Imhoff GW Kluin-Nelemans HC Boerma EJ. Double-Hit B-Cell Lymphomas. Blood (2011) 117:2319–31. doi: 10.1182/blood-2010-09-297879 21119107

[9] Riedell P Smith S. Double Hit and Double Expressors in Lymphoma: Definition and Treatment. Cancer (2018) 124:4622–32. doi: 10.1002/cncr.31646 30252929

[10] Miyazaki K. Treatment of Diffuse Large B-Cell Lymphoma. J Clin Exp Hematop (2016) 56:79–88. doi: 10.3960/jslrt.56.79 27980306 PMC6144206

[11] Alizadeh AA Gentles AJ Alencar AJ Liu CL Kohrt HE Houot R. Prediction of Survival in Diffuse Large B-Cell Lymphoma Based on the Expression of 2 Genes Reflecting Tumor and Microenvironment. Blood (2011) 118:1350–8. doi: 10.1182/blood-2011-03-345272 PMC3152499 21670469

[12] Zelenetz AD Abramson JS Advani RH Andreadis CB Byrd JC Czuczman MS. NCCN Clinical Practice Guidelines in Oncology: Non-Hodgkin’s Lymphomas. J Natl Compr Canc Netw (2010) 8:288–334. doi: 10.6004/jnccn.2010.0021 20202462

[13] Gutiérrez-García G Cardesa-Salzmann T Climent F González-Barca E Mercadal S Mate JL. Gene-Expression Profiling and Not Immunophenotypic Algorithms Predicts Prognosis in Patients With Diffuse Large B-Cell Lymphoma Treated With Immunochemotherapy. Blood (2011) 117:4836–43. doi: 10.1182/blood-2010-12-322362 21441466

[14] Yin Z Dong C Jiang K Xu Z Li R Guo K. Heterogeneity of Cancer-Associated Fibroblasts and Roles in the Progression, Prognosis, and Therapy of Hepatocellular Carcinoma. J Hematol Oncol (2019) 12:101. doi: 10.1186/s13045-019-0782-x 31547836 PMC6757399

[15] Zhang D He W Wu C Tan Y He Y Xu B. Scoring System for Tumor-Infiltrating Lymphocytes and Its Prognostic Value for Gastric Cancer. Front Immunol (2019) 10:71. doi: 10.3389/fimmu.2019.00071 30761139 PMC6361780

[16] Autio M Leivonen SK Brück O Mustjoki S Jørgensen JM Karjalainen-Lindsberg ML. Immune Cell Constitution in the Tumor Microenvironment Predicts the Outcome in Diffuse Large B-Cell Lymphoma. Haematologica (2020) 106(3):718–29. doi: 10.3324/haematol.2019.243626 PMC7927991 32079690

[17] Cioroianu AI Stinga PI Sticlaru L Cioplea MD Nichita L Popp C. Tumor Microenvironment in Diffuse Large B-Cell Lymphoma: Role and Prognosis. Anal Cell Pathol (Amst) (2019) 2019:8586354. doi: 10.1155/2019/8586354 31934533 PMC6942707

[18] Opinto G Vegliante MC Negri A Skrypets T Loseto G Pileri SA. The Tumor Microenvironment of DLBCL in the Computational Era. Front Oncol (2020) 10:351. doi: 10.3389/fonc.2020.00351 32296632 PMC7136462

[19] Xie Z Li M Hong H Xu Q He Z Peng Z. Expression of N(6)-Methyladenosine (M(6)A) Regulators Correlates With Immune Microenvironment Characteristics and Predicts Prognosis in Diffuse Large Cell Lymphoma (DLBCL). Bioengineered (2021) 12:6115–33. doi: 10.1080/21655979.2021.1972644 PMC8806613 34482808

[20] Feng P Li H Pei J Huang Y Li G. Identification of a 14-Gene Prognostic Signature for Diffuse Large B Cell Lymphoma (DLBCL). Front Genet (2021) 12:625414. doi: 10.3389/fgene.2021.625414 PMC7902938 33643388

[21] Luo C Nie H Yu L. Identification of Aging-Related Genes Associated With Prognostic Value and Immune Microenvironment Characteristics in Diffuse Large B-Cell Lymphoma. Oxid Med Cell Longev (2022) 2022:3334522. doi: 10.1155/2022/3334522 PMC8777392 35069971

[22] He R Zuo S. A Robust 8-Gene Prognostic Signature for Early-Stage Non-Small Cell Lung Cancer. Front Oncol (2019) 9:693. doi: 10.3389/fonc.2019.00693 31417870 PMC6684755

[23] Friedman J Hastie T Tibshirani R. Regularization Paths for Generalized Linear Models *via* Coordinate Descent. J Stat Softw (2010) 33:1–22. doi: 10.18637/jss.v033.i01 20808728 PMC2929880

[24] Newman A Liu C Green M Gentles A Feng W Xu Y. Robust Enumeration of Cell Subsets From Tissue Expression Profiles. Nat Methods (2015) 12:453–7. doi: 10.1038/nmeth.3337 PMC4739640 25822800

[25] Bindea G Mlecnik B Tosolini M Kirilovsky A Waldner M Obenauf A. Spatiotemporal Dynamics of Intratumoral Immune Cells Reveal the Immune Landscape in Human Cancer. Immunity (2013) 39:782–95. doi: 10.1016/j.immuni.2013.10.003 24138885

[26] Iasonos A Schrag D Raj GV Panageas KS. How to Build and Interpret a Nomogram for Cancer Prognosis. J Clin Oncol (2008) 26:1364–70. doi: 10.1200/JCO.2007.12.9791 18323559

[27] Chapuy B Stewart C Dunford AJ Kim J Kamburov A Redd RA. Molecular Subtypes of Diffuse Large B Cell Lymphoma Are Associated With Distinct Pathogenic Mechanisms and Outcomes. Nat Med (2018) 24:679–90. doi: 10.1038/s41591-018-0016-8 PMC6613387 29713087

[28] Crombie J. Classifying DLBCL Subtypes for Optimal Treatment. Oncol (Williston Park) (2019) 33(10):686504. 31661151

[29] Link B. Foreseeing What Is to Happen in DLBCL. Blood (2020) 135:2014–5. doi: 10.1182/blood.2020005678 32497224

[30] Schmitz R Wright G Huang D Johnson C Phelan J Wang J. Genetics and Pathogenesis of Diffuse Large B-Cell Lymphoma. N Engl J Med (2018) 378:1396–407. doi: 10.1056/NEJMoa1801445 PMC6010183 29641966

[31] Han H Fan G Song S Jiang Y Qian C Zhang W. piRNA-30473 Contributes to Tumorigenesis and Poor Prognosis by Regulating M6a RNA Methylation in DLBCL. Blood (2021) 137:1603–14. doi: 10.1182/blood.2019003764 32967010

[32] Ye Z Zou S Niu Z Xu Z Hu Y. A Novel Risk Model Based on Lipid Metabolism-Associated Genes Predicts Prognosis and Indicates Immune Microenvironment in Breast Cancer. Front Cell Dev Biol (2021) 9:691676. doi: 10.3389/fcell.2021.691676 34195202 PMC8236894

[33] Cao R Yuan L Ma B Wang G Tian Y. Immune-Related Long Non-Coding RNA Signature Identified Prognosis and Immunotherapeutic Efficiency in Bladder Cancer (BLCA). Cancer Cell Int (2020) 20:276. doi: 10.1186/s12935-020-01362-0 32607061 PMC7320553

[34] Zhu G Xia H Tang Q Bi F. An Epithelial-Mesenchymal Transition-Related 5-Gene Signature Predicting the Prognosis of Hepatocellular Carcinoma Patients. Cancer Cell Int (2021) 21:166. doi: 10.1186/s12935-021-01864-5 33712026 PMC7953549

[35] Chen J Yu Y Li H Hu Q Chen X He Y. Long Non-Coding RNA PVT1 Promotes Tumor Progression by Regulating the miR-143/HK2 Axis in Gallbladder Cancer. Mol Cancer (2019) 18:33. doi: 10.1186/s12943-019-0947-9 PMC6397746 30825877

[36] Mathupala SP Ko YH Pedersen PL. Hexokinase II: Cancer’s Double-Edged Sword Acting as Both Facilitator and Gatekeeper of Malignancy When Bound to Mitochondria. Oncogene (2006) 25:4777–86. doi: 10.1038/sj.onc.1209603 PMC3385868 16892090

[37] Fan K Fan Z Cheng H Huang Q Yang C Jin K. Hexokinase 2 Dimerization and Interaction With Voltage-Dependent Anion Channel Promoted Resistance to Cell Apoptosis Induced by Gemcitabine in Pancreatic Cancer. Cancer Med (2019) 8:5903–15. doi: 10.1002/cam4.2463 PMC6792491 31426130

[38] Bhalla K Jaber S Nahid MN Underwood K Beheshti A Landon A. Role of Hypoxia in Diffuse Large B-Cell Lymphoma: Metabolic Repression and Selective Translation of HK2 Facilitates Development of DLBCL. Sci Rep (2018) 8:744–. doi: 10.1038/s41598-018-19182-8 PMC5768748 29335581

[39] Sang H Li T Li H Liu J. Down-Regulation of Gab1 Inhibits Cell Proliferation and Migration in Hilar Cholangiocarcinoma. PloS One (2013) 8:e81347–e. doi: 10.1371/journal.pone.0081347 PMC3842939 24312291

[40] Liu H Li G Zeng W Zhang P Fan F Tu Y. Combined Detection of Gab1 and Gab2 Expression Predicts Clinical Outcome of Patients With Glioma. Med Oncol (2014) 31:77. doi: 10.1007/s12032-014-0077-6 24998422

[41] Chang RX Cui AL Dong L Guan SP Jiang LY Miao CX. Overexpression of RASAL1 Indicates Poor Prognosis and Promotes Invasion of Ovarian Cancer. Open Life Sci (2019) 14:133–40. doi: 10.1515/biol-2019-0015 PMC7874762 33817145

[42] Basagaña X Barrera-Gómez J. Reflection on Modern Methods: Visualizing the Effects of Collinearity in Distributed Lag Models. Int J Epidemiol (2021) 51(1):334–44. doi: 10.1093/ije/dyab179 34458914

[43] Sarkozy C Sehn L. Management of Relapsed/Refractory DLBCL. Best Pract Res Clin Haematol (2018) 31:209–16. doi: 10.1016/j.beha.2018.07.014 30213390

[44] Aggarwal A Rafei H Alakeel F Finianos AN Liu M-L El-Bahesh E. Outcome of Patients With Double-Expressor Lymphomas (DELs) Treated With R-CHOP or R-EPOCH. Blood (2016) 128:5396. doi: 10.1182/blood.V128.22.5396.5396

[45] Hu S Xu-Monette Z Tzankov A Green T Wu L Balasubramanyam A. MYC/BCL2 Protein Coexpression Contributes to the Inferior Survival of Activated B-Cell Subtype of Diffuse Large B-Cell Lymphoma and Demonstrates High-Risk Gene Expression Signatures: A Report From The International DLBCL Rituximab-CHOP Consortium Program. Blood (2013) 121:4021–31; quiz 250. doi: 10.1182/blood-2012-10-460063 23449635 PMC3709650

[46] Martelli M Ferreri AJM Agostinelli C Di Rocco A Pfreundschuh M Pileri SA. Diffuse Large B-Cell Lymphoma. Crit Rev Oncol/Hematol (2013) 87:146–71. doi: 10.1016/j.critrevonc.2012.12.009 23375551

[47] Papageorgiou S Thomopoulos T Katagas I Bouchla A Pappa V. Prognostic Molecular Biomarkers in Diffuse Large B-Cell Lymphoma in the Rituximab Era and Their Therapeutic Implications. Ther Adv Hematol (2021) 12:20406207211013987. doi: 10.1177/20406207211013987 34104369 PMC8150462

[48] Klyuchnikov E Bacher U Kroll T Shea T Lazarus H Bredeson C. Allogeneic Hematopoietic Cell Transplantation for Diffuse Large B Cell Lymphoma: Who, When and How? Bone Marrow Transplant (2014) 49:1–7. doi: 10.1038/bmt.2013.72 23708703

[49] Lenz G Wright G Dave S Xiao W Powell J Zhao H. Stromal Gene Signatures in Large-B-Cell Lymphomas. N Engl J Med (2008) 359:2313–23. doi: 10.1056/NEJMoa0802885 PMC9103713 19038878

[50] Mueller C Boix C Kwan W Daussy C Fournier E Fridman W. Critical Role of Monocytes to Support Normal B Cell and Diffuse Large B Cell Lymphoma Survival and Proliferation. J Leukocyte Biol (2007) 82:567–75. doi: 10.1189/jlb.0706481 17575267

[51] Chang K Huang G Jones D Lin Y. Distribution Patterns of Dendritic Cells and T Cells in Diffuse Large B-Cell Lymphomas Correlate With Prognoses. Clin Cancer Res (2007) 13:6666–72. doi: 10.1158/1078-0432.CCR-07-0504 18006767

[52] Staiger A Altenbuchinger M Ziepert M Kohler C Horn H Huttner M. A Novel Lymphoma-Associated Macrophage Interaction Signature (LAMIS) Provides Robust Risk Prognostication in Diffuse Large B-Cell Lymphoma Clinical Trial Cohorts of the DSHNHL. Leukemia (2020) 34:543–52. doi: 10.1038/s41375-019-0573-y 31530861

[53] Kridel R Steidl C Gascoyne R. Tumor-Associated Macrophages in Diffuse Large B-Cell Lymphoma. Haematologica (2015) 100:143–5. doi: 10.3324/haematol.2015.124008 PMC4803134 25638802

