# Supplementary figures and images for: Identification and Validation of a Prognostic Prediction Model in Diffuse Large B-Cell Lymphoma
Source: Front Endocrinol (Lausanne). 2022 Apr 14;13:846357. doi: 10.3389/fendo.2022.846357 (PMC9048048; doi:10.3389/fendo.2022.846357)

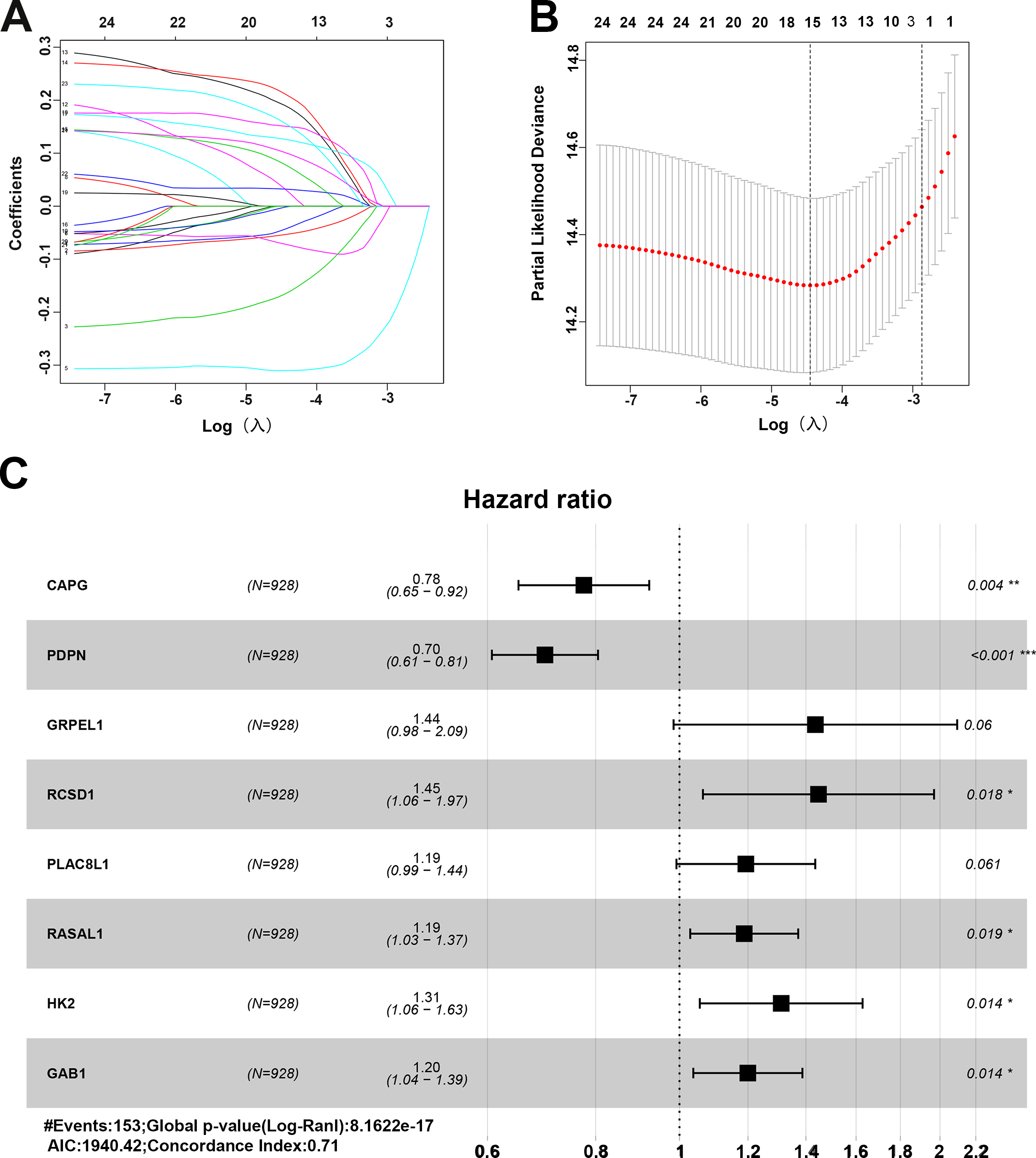

Supplement: Supplementary Figure 1 — Selection of genes for constructing predicting model. (A) LASSO coefficient profiles of 24 genes. (B) Partial likelihood deviance plot. (C) Forest plot shows the multivariable Cox regression analysis of eight genes. [file Image_1.tif]

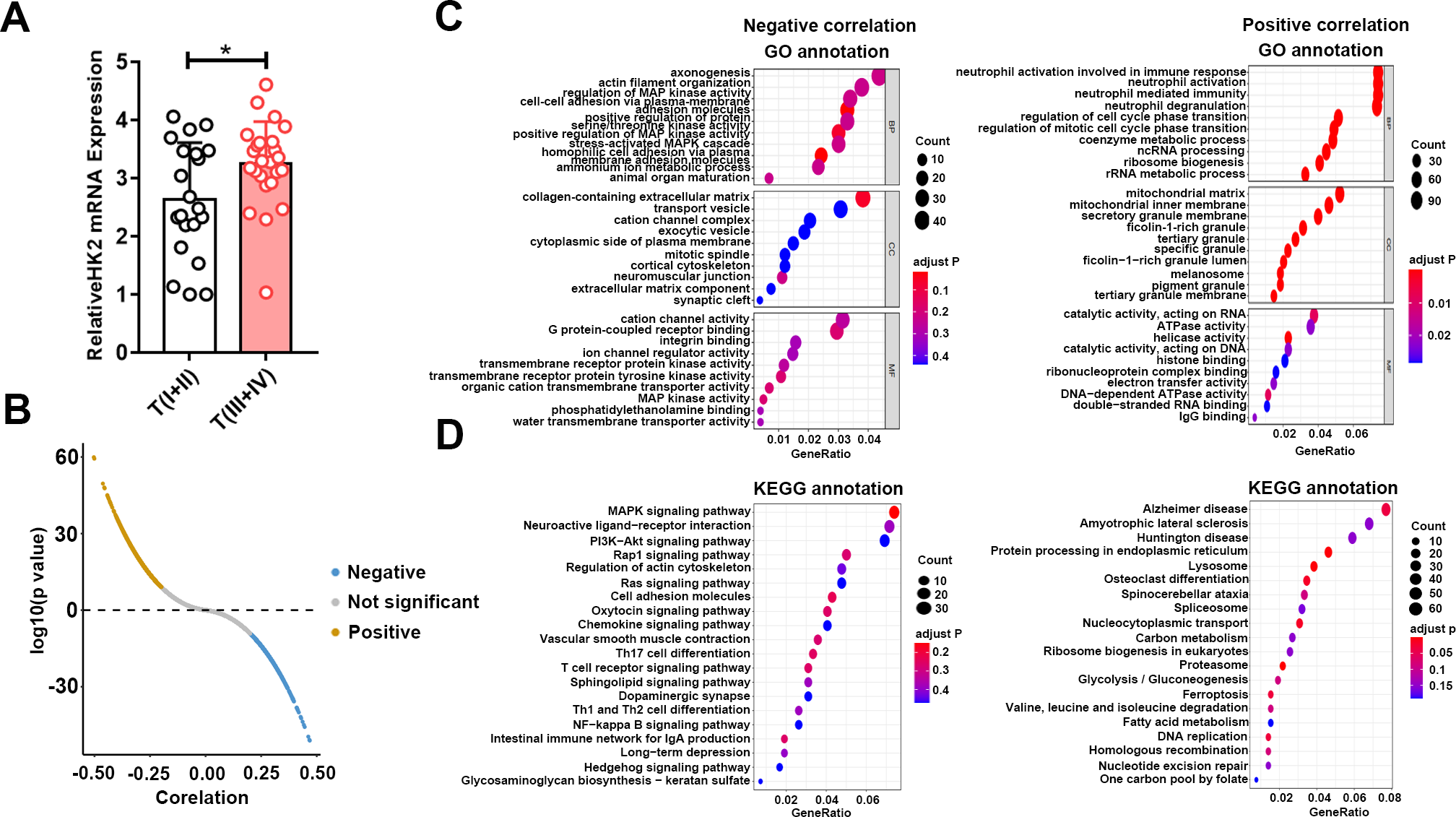

Supplement: Supplementary Figure 2 — Exploring function of HK2. (A) RT-PCR analysis showing HK2 expression stage I+II and III+IV. (B) Correlation analysis of HK2. (C) GO enrichment analysis of negative and positive correlated genes. (D) KEGG enrichment analysis of negative and positive correlated genes. [file Image_2.tif]

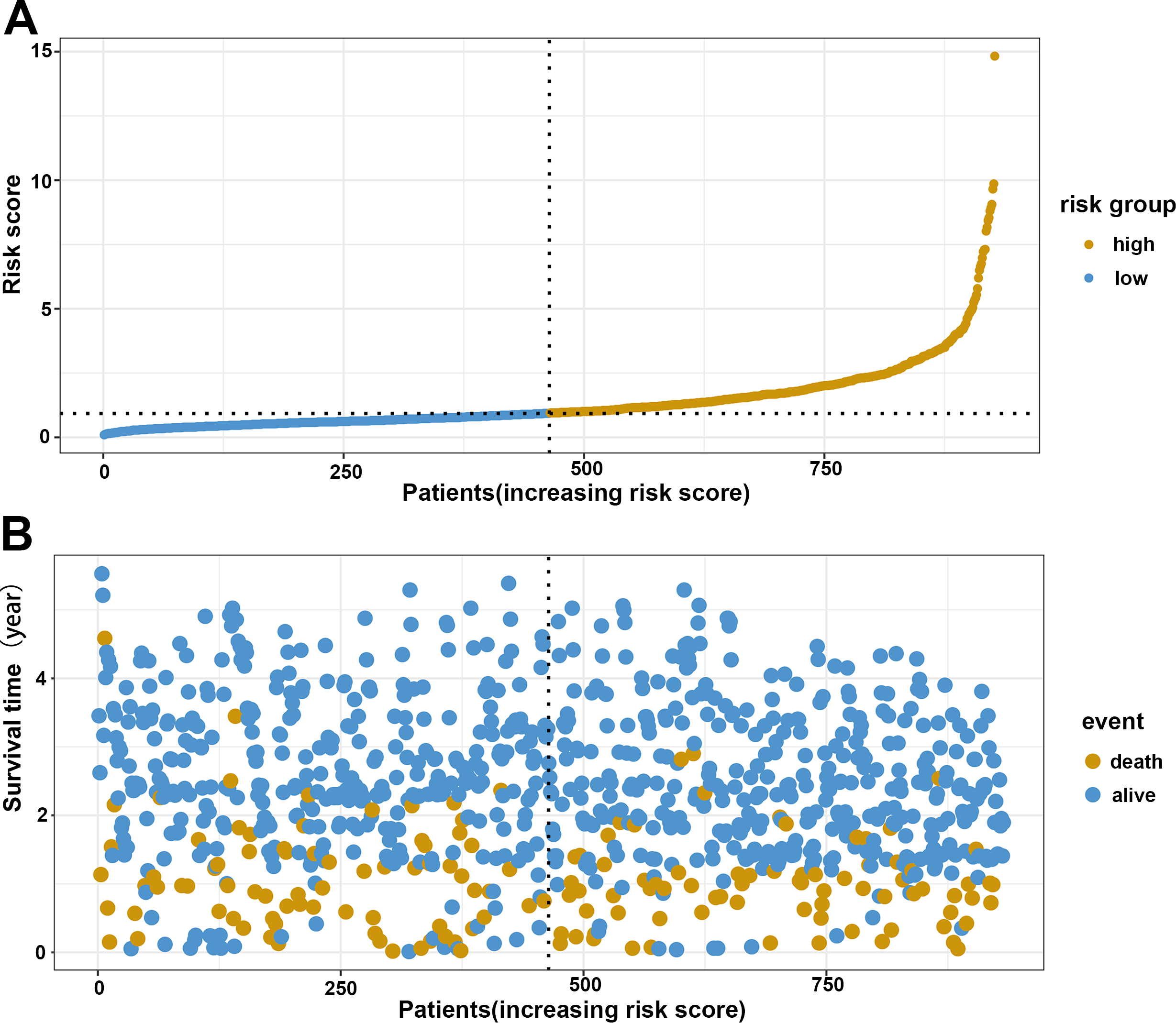

Supplement: Supplementary Figure 3 — Survival information of the patients between high- and low-risk group. (A) The correlation of risk score and patient numbers. (B) The correlation of risk score and survival time. [file Image_3.tif]

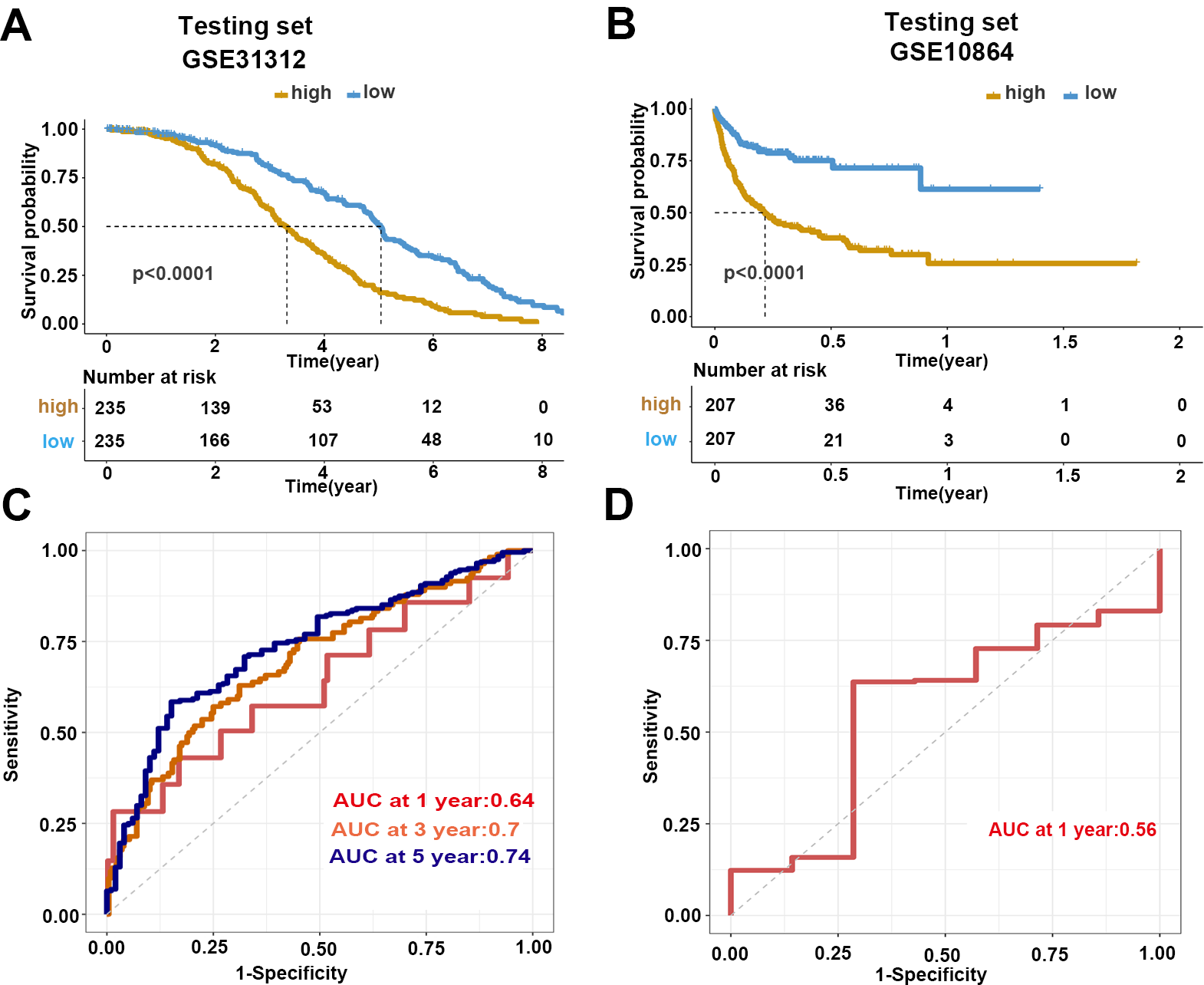

Supplement: Supplementary Figure 4 — Validation of prediction model in testing set. (A, B) Kaplan-Meier analysis between high and low risk group in GSE31312 and GSE10864 datasets. (C, D) ROC curve analysis of GSE31312 and GSE10864 datasets. [file Image_4.tif]

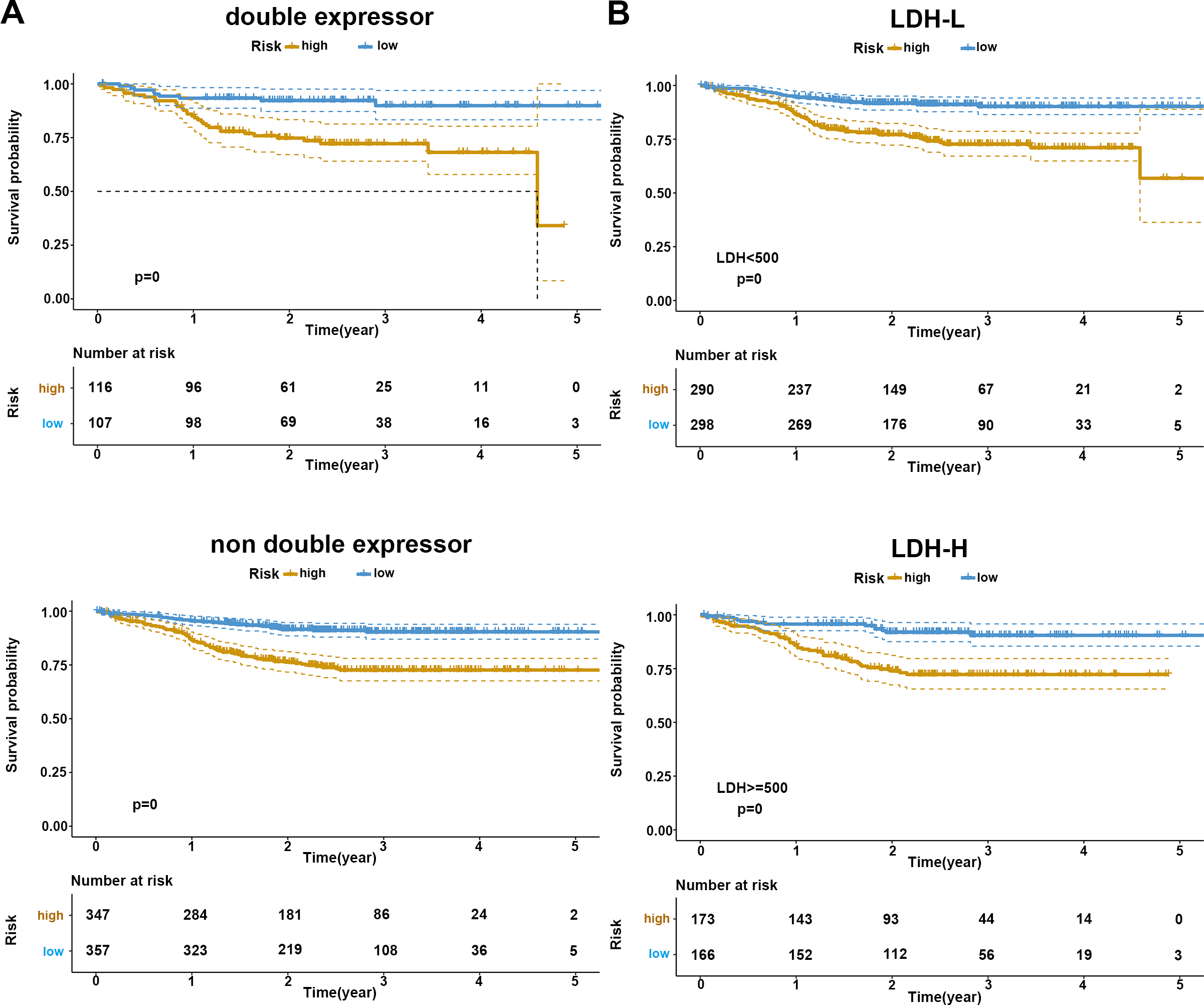

Supplement: Supplementary Figure 5 — KM survival stratifcation analyses in GSE117556 dataset. (A) MYC/BCL2 expression. (B) LDH level. [file Image_5.tif]

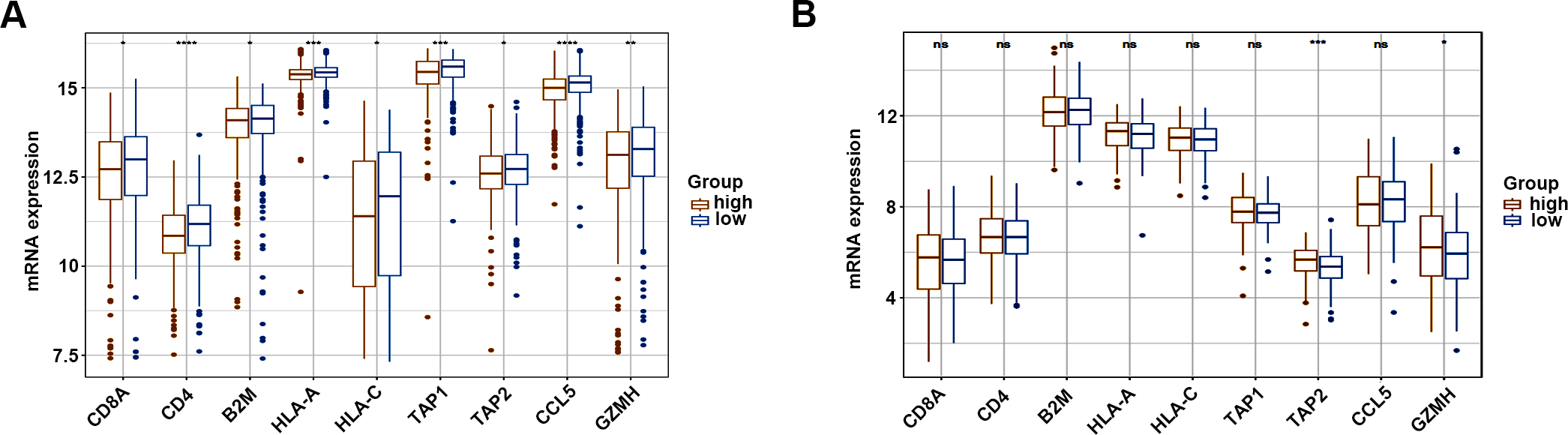

Supplement: Supplementary Figure 6 — Expression of immune-related genes in high and low risk group. (A, B) Boxplot showed difference of immune-related genes in high and low risk group in GSE117556 and TCGA-NCICCR dataset, respectively. [file Image_6.tif]
