# Supplementary material for: Identification and Validation of a Prognostic Prediction Model in Diffuse Large B-Cell Lymphoma
Source: Front Endocrinol (Lausanne). 2022 Apr 14;13:846357. doi: 10.3389/fendo.2022.846357 (PMC9048048; doi:10.3389/fendo.2022.846357)
Supplement: Supplementary file 7 [file Table_1.docx]

**Supplement Table 1: Clinicopathological features of 45 DLBCL patients from the First Affiliated Hospital of Zhengzhou University**

| Characteristic | n |
| --- | --- |
| Age（years） |  |
| ≥60 | 31 |
| <60 | 14 |
| Gender |  |
| Male | 24 |
| Female | 21 |
| COO class |  |
| ABC DLBCL | 19 |
| GCB DLBCL | 25 |
| Unclassified DLBCL | 1 |
| TNM stage |  |
| Stage 1 | 9 |
| Stage 2 | 13 |
| Stage 3 | 11 |
| Stage 4 | 12 |
|  |  |
|  |  |
