# Supplementary material for: Identification and Validation of a Prognostic Prediction Model in Diffuse Large B-Cell Lymphoma
Source: Front Endocrinol (Lausanne). 2022 Apr 14;13:846357. doi: 10.3389/fendo.2022.846357 (PMC9048048; doi:10.3389/fendo.2022.846357)
Supplement: Supplementary file 8 [file Table_2.docx]

| Gene | Forward | Reverse |
| --- | --- | --- |
| CAPG | 5' –GGGGACTCCTACCTAGTGCTG-3' | 5' –CACCACCTTCCTGGTACTTGA-3' |
| PDPN | 5' -AACCAGCGAAGACCGCTATAA-3' | 5' –CGAATGCCTGTTACACTGTTGA-3' |
| GRPEL1 | 5' -TCCTGCTTTGGCGTTGTCTC-3' | 5' –CTGCCGTAAGTTCTCAGTGTC-3' |
| RCSD1 | 5' -AGCCAGTAAACCAACCCGAAG-3' | 5' –CTCGCATTGGGTGGTGATT-3' |
| PLAC8L1 | 5' -CAGTGGCAGGACGACAATCA-3' | 5' –ACACTCAAGACACATAGGACAGA-3' |
| RASAL1 | 5' -CAGCTCCCTGAATGTTCGC-3' | 5' –TCCTCATCCAGCACGTAGAAG-3' |
| HK2 | 5' -GAGCCACCACTCACCCTACT-3' | 5' –CCAGGCATTCGGCAATGTG-3' |
| GAB1 | 5' -GATGGTTCGTGTTACGCAGTG-3' | 5' –CGCTGTCTGCTACCAAGTAGAA-3' |

**Supplementary table2：Primers of genes used in this study**
